# Development and Characterization of an Allergic Asthma Rat Model for Interventional Studies

**DOI:** 10.3390/ijms21113841

**Published:** 2020-05-28

**Authors:** Marta Périz, Francisco J. Pérez-Cano, Maria J. Rodríguez-Lagunas, Trinitat Cambras, Santiago Pastor-Soplin, Iván Best, Margarida Castell, Malén Massot-Cladera

**Affiliations:** 1 Secció de Fisiologia, Departament de Bioquímica i Fisiologia, Facultat de Farmàcia i Ciències de l’Alimentació, Universitat de Barcelona (UB), 08028 Barcelona, Spain; mperiz@ub.edu (M.P.); franciscoperez@ub.edu (F.J.P.-C.); mjrodriguez@ub.edu (M.J.R.-L.); cambras@ub.edu (T.C.); malen.massot@ub.edu (M.M.-C.); 2 Institut de Recerca en Nutrició i Seguretat Alimentària (INSA-UB), UB, 08921 Santa Coloma de Gramenet, Spain; 3 Programa Cacao, Ingeniería Agroforestal, Facultad de Ciencias Ambientales, Universidad Científica del Sur, 15842 Lima, Peru; spastor@ucientifica.edu.pe (S.P.-S.); ibest@usil.edu.pe (I.B.); 4 Unidad de Investigación en Nutrición, Salud, Alimentos Funcionales y Nutracéuticos, Universidad San Ignacio de Loyola, 15024 Lima, Peru

**Keywords:** body temperature, bronchoalveolar lavage fluid, Brown Norway rats, IgE, intranasal challenge, leukotriene, motor activity, ovalbumin

## Abstract

Allergic asthma is one of the most common chronic diseases of the airways, however it still remains underdiagnosed and hence undertreated. Therefore, an allergic asthma rat model would be useful to be applied in future therapeutic strategy studies. The aim of the present study was to develop an objective model of allergic asthma in atopic rats that allows the induction and quantification of anaphylactic shock with quantitative variables. Female Brown Norway rats were intraperitoneally sensitized with ovalbumin (OVA), alum and *Bordetella pertussis* toxin and boosted a week later with OVA in alum. At day 28, all rats received an intranasal challenge with OVA. Anaphylactic response was accurately assessed by changes in motor activity and body temperature. Leukotriene concentration was determined in the bronchoalveolar lavage fluid (BALF), and total and IgE anti-OVA antibodies were quantified in blood and BALF samples. The asthmatic animals’ motility and body temperature were reduced after the shock for at least 20 h. The asthmatic animals developed anti-OVA IgE antibodies both in BALF and in serum. These results show an effective and relatively rapid model of allergic asthma in female Brown Norway rats that allows the quantification of the anaphylactic response.

## 1. Introduction

Allergic asthma is a chronic inflammatory disorder of the airways, caused by an immunological-mediated hypersensitivity reaction [1]. A range of underlying mechanisms causes airway inflammation that involves variable airflow limitation and respiratory symptoms. Wheezing, shortness of breath, chest tightness and cough are the most characteristic asthma features [2]. The condition usually starts in childhood, although it can also develop in adulthood, and affects people of all ages. There is currently no cure, but treatment can help control the symptoms [1]. Although type 2-driven inflammation is key in allergic asthma, the pathophysiology is complex and involves several pathogenic pathways that allow the definition of disease endotypes [3]. Asthma is one of the most common chronic, non-communicable diseases, and affects around 270 million people worldwide [4]. Nevertheless, asthma still remains underdiagnosed and hence undertreated, producing quality of life and lifestyle disruptions and creating a burden on families, societies and countries [5].

Animal models of asthma have been developed in mice and rats [6,7,8,9,10,11,12], as they share many features of human allergic asthma [13]. Transgenic technology has been applied in mouse models which allowed the identification of particular genes involved in the pathology of asthma [14]. Nevertheless, the main symptoms of asthma, such as airway hyperresponsiveness and inflammation, can be more easily reproduced in rats than in mice [10]. In addition, rats are easier to handle and larger than mice which permit the collection of higher amounts of samples. For these reasons, rat models of asthma are increasing in importance [7]. However, rat strains such as Wistar, Sprague Dawley, Fisher and Lewis do not always develop an allergic response with IgE production [7,15]. Despite this fact, asthma models in Wistar and Sprague Dawley rats have been reported [12,16]. In contrast, the Brown Norway rat is an atopic strain prone to produce IgE responses after allergen sensitization [17,18,19]; hence, it is a suitable strain for studying allergic asthma. Although a variety of allergens have been used for allergic sensitization, ovalbumin (OVA) is one of the most commonly used [7,11,12,20]. Several types of models have been developed varying the OVA-sensitization protocol in terms of the route of administration, the adjuvant, the number of sensitizations and the number of challenges carried out [11,12]. The aim of the current study was to develop an objective model of allergic asthma in Brown Norway rats that allows the induction and quantification of an anaphylactic shock with quantitative variables. This model could be applied in future studies of therapeutic strategies aimed at preventing or decreasing allergic asthma symptoms. To carry out our investigation, two experimental procedures were designed. Firstly, a preliminary design sought to address whether *Bordetella pertussis* toxin (Bpt) was required for allergy induction and whether the conditions tested were sufficient to induce an anaphylactic response by intranasal (i.n.) route. After this, a definitive study was carried out, focused on establishing objective variables to quantify the anaphylactic response.

## 2. Results

### 2.1. Preliminary Study

#### 2.1.1. Serum Anti-OVA IgE Antibodies

**Figure 1:**
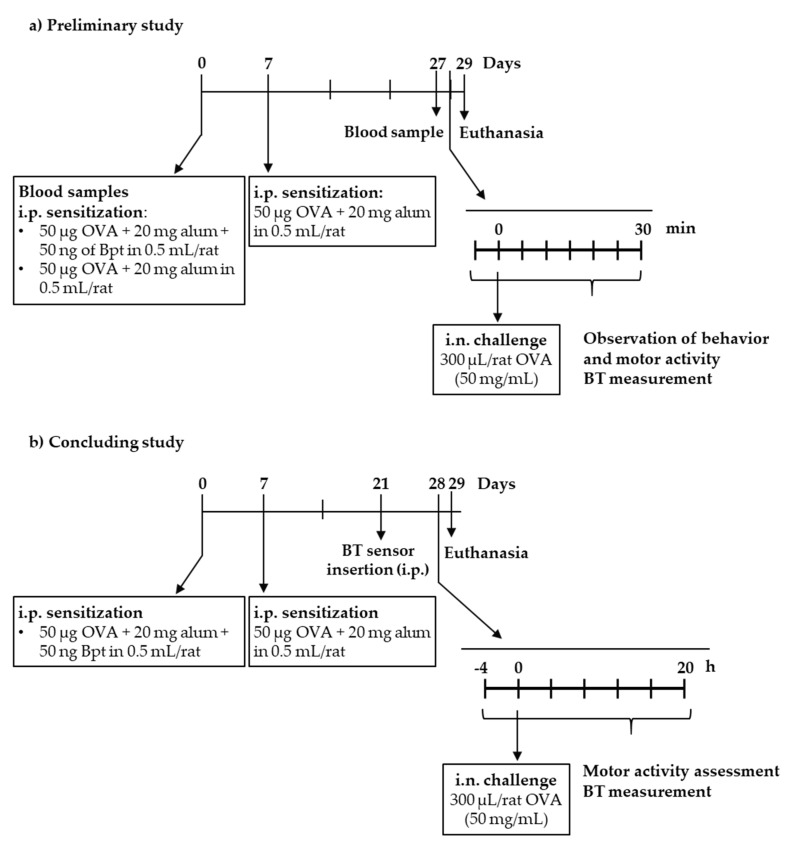
Experimental designs followed in the (**a**) preliminary study and (**b**) the definitive study. Bpt: *Bordetella pertussis* toxin; BT: body temperature; i.n.: intranasal; i.p.: intraperitoneal; OVA: ovalbumin.

**Figure 2:**
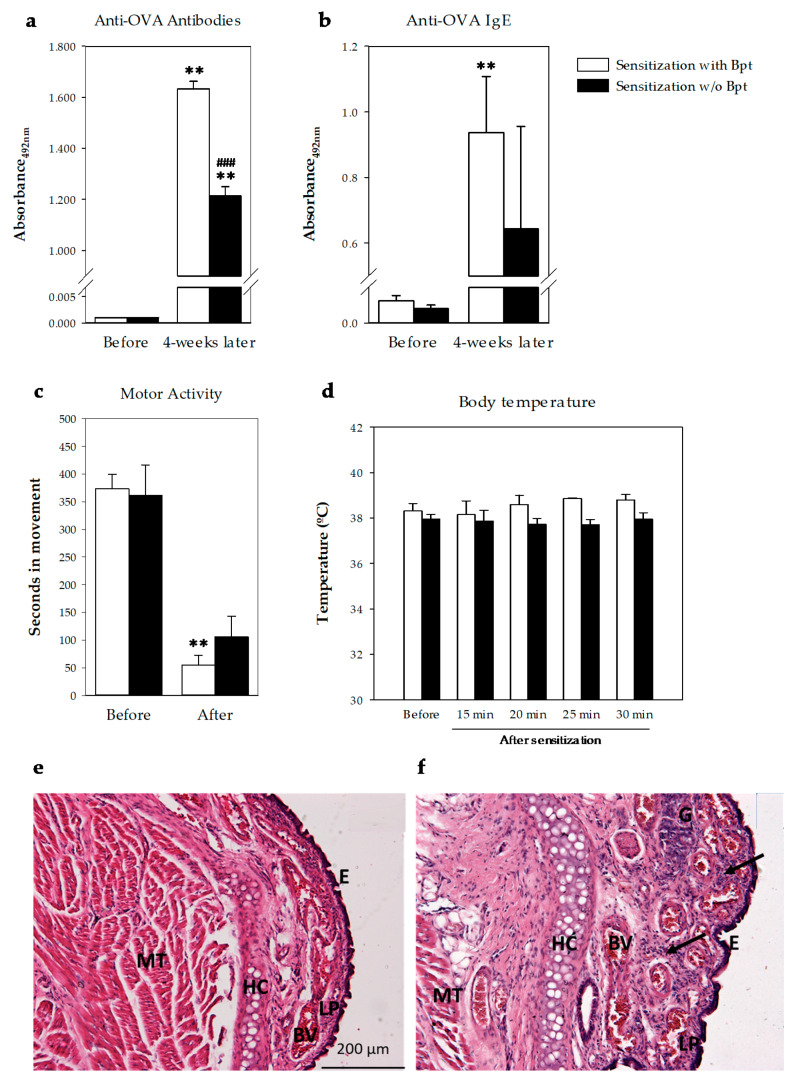
Effect of sensitization with (white bars) or without (w/o, black bars) Bpt on (**a**) serum total anti-OVA antibodies and (**b**) serum anti-OVA IgE before and 4 weeks after sensitization. Changes on (**c**) motor activity and (**d**) body temperature before and after the challenge with i.n. OVA (50 mg/mL) in sensitized rats with (white bars) or without (black bars) Bpt. Results are expressed as mean ± standard error (*N* = 4) of absorbance units. Significant differences: ** *p* < 0.01 vs. basal values; ### *p* < 0.001 vs. sensitization with Bpt. Changes in respiratory airway 24 h after the i.n. challenge (**f**) in comparison with non-challenged animals (**e**). Representative histological images of respiratory mucosa of nasal sections stained with hematoxylin and eosin, 100×, scale bar = 200 µm. E, epithelium; LP, lamina propria; BV, blood vessels; HC, hyaline cartilage; MT, muscle tissue; G, gland. Arrow indicates leukocyte infiltrate.

The first experiments were carried out in order to establish whether Bpt was needed to induce an IgE-mediated allergy in the animals. Two groups of rats were studied, one with the presence of Bpt in the first sensitization and one group without (Figure 1a). At day 27 from the first sensitization, all rats developed anti-OVA antibodies, some of them belonging to the IgE isotype (Figure 2a,b). Considering the mean of basal values plus two standard deviations as the negative cut-off, 100% of the animals developed anti-OVA antibodies and anti-OVA IgE. The group immunized with Bpt presented the highest total anti-OVA antibody levels (*p* < 0.0001 vs. basal levels and those from the non-Bpt immunized group) with an increase in anti-OVA IgE antibody production (*p* < 0.01 vs. basal levels).

#### 2.1.2. Intranasal-Induced Shock Quantification

At day 29, the rats, sensitized or not with Bpt, were challenged by i.n. administration of either 5 or 50 mg/mL OVA. Motor activity was assessed in a blinded manner by quantifying the time each animal spent moving in a video recorded during the first 10 min after the challenge and comparing the results with its own basal movements (i.e., before the challenge) during the same period of time (Figure 2c). Although both types of sensitizations affected the rats’ movements after the i.n. challenge, the decrease was only significant in the Bpt-sensitized animals with respect to basal conditions. Moreover, the behavior observation revealed the appearance of wheezing only in the Bpt-sensitized animals that had been challenged with the highest dose of OVA.

Body temperature was also measured before and every 5 min from 15 min to 30 min after the i.n. challenge (Figure 2d). No significant changes were observed during the studied period.

#### 2.1.3. Respiratory Airway Histology

Nasal respiratory mucosa of animals sensitized with Bpt and challenged with i.n. OVA exhibited mucosal thickening in the nasal subepithelial, with strong leukocytic cell infiltration in the lamina propria compared to unchallenged animals (Figure 2e,f). Moreover, submucosal gland hypertrophy was also observed in challenged animals (Figure 2f).

The results of the preliminary study allowed us to conclude that the Brown Norway rats immunized with Bpt became more sensitized than those that did not receive it and that 4 weeks from the first sensitization was enough to induce an anaphylactic shock by i.n. route. This shock induced behavioral changes which, to be objectively quantified, required better approaches such as those applied in the second and definitive study.

### 2.2. Definitive Study

In the second design, rats sensitized with OVA together with Bpt and boosted one week later were challenged by i.n. route with OVA on day 28 (Figure 1b). Concurrently, a group of healthy rats was also challenged.

#### 2.2.1. Motor Activity

**Figure 3:**
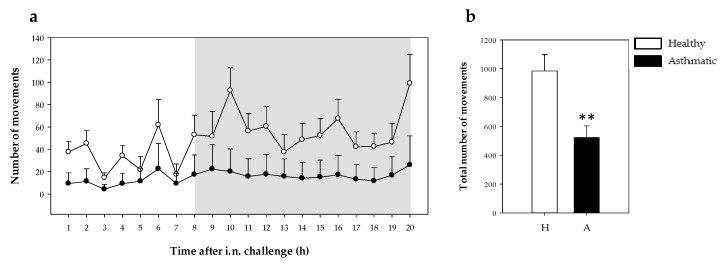
Motor activity during 20 h after the i.n. challenge. (**a**) Motor activity assessed every hour (the shadow period corresponds to darkness) in healthy (H, white symbol) and asthmatic (A, black symbol) rats. Statistical differences not included. (**b**) Total number of movements in healthy (H, white bars) and asthmatic (A, black bars) 20 h after the challenge. Results are expressed as mean ± standard error (*N* = 9). Significant differences: ** *p* < 0.01 vs. healthy animals.

Motor activity was measured at 1 h intervals from 4 h before the challenge until 20 h after. Before challenging, basal motor activity did not differ between healthy and sensitized rats. The number of movements recorded for 4 h was 175.22 ± 33.32 vs. 127.89 ± 27.44 (mean ± standard error for nine animals) in the healthy group and asthmatic group, respectively. However, the i.n. challenge with OVA decreased the movements of asthmatic rats as observed in the 20 h period studied after shock induction (Figure 3a). Moreover, the number of movements during darkness in asthmatic rats did not show the characteristic increase in motor activity present in healthy rats. The differences among the groups’ movements in the same period of time was also observed when the total number of movements was calculated (*p* = 0.008) (Figure 3b).

#### 2.2.2. Temperature

**Figure 4:**
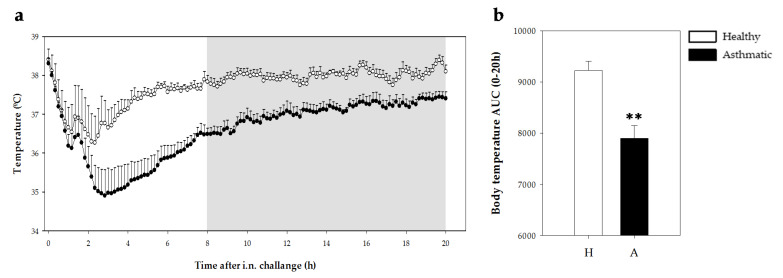
Body temperature 20 h after the i.n. challenge. (**a**) Body temperature assessed every 10 min (grey period corresponds to the dark period) in healthy (H, white bars) and asthmatic (A, black bars) rats. Statistical differences not included. (**b**) Area under the curve (AUC) of body temperature changes (from 34 °C) in the same period. Results are expressed as mean ± standard error (*N* = 9). Significant differences: ** *p* < 0.01 vs. healthy animals.

Body temperature was measured by means of a data logger during 20 h after the challenge in healthy and asthmatic groups. There were no differences in body temperature between healthy and sensitized rats in the 4 h period before the challenge (37.80 °C ± 0.10 °C vs. 37.64 °C ± 0.05 °C; mean ± standard error for nine animals in healthy and sensitized groups, respectively). The i.n. challenge with OVA induced a reduction in body temperature in both healthy and asthmatic rats in the first 3 h post-challenge (Figure 4a). The body temperature of healthy rats decreased by 1.9 °C ± 0.6 °C (mean ± standard error) whereas asthmatic rats displayed a reduction of 3.2 °C ± 0.7 °C. In addition, the asthmatic group showed lower body temperature than healthy rats for the 20 h period studied as observed in the time course (Figure 4a) as well as in the area under the curve (AUC) (*p* = 0.001; Figure 4b).

#### 2.2.3. Serum Anti-OVA Antibodies.

**Figure 5:**
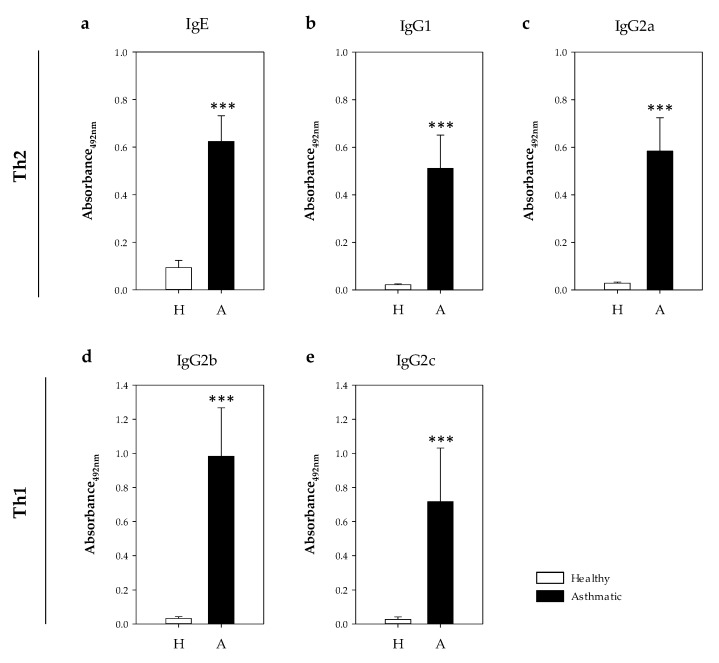
Concentration of serum OVA-specific antibodies 24 h post-challenge. (**a**) IgE, (**b**) IgG1, (**c**) IgG2a, (**d**) IgG2b and (**e**) IgG2c in healthy (H, white bars) and asthmatic (A, black bars) rats. Results are expressed as mean ± standard error (*N* = 9). Significant differences: *** *p* < 0.001 vs. healthy animals.

Specific anti-OVA antibodies belonging to IgE and IgG isotypes were quantified in serum samples collected the day after the i.n. challenge (Figure 5). In comparison to healthy rats, asthmatic animals showed significant levels of specific antibodies belonging to IgE, IgG1 and IgG2a isotypes (Figure 5a–c), which were associated with a Th2 response [17,21,22], as well as those belonging to IgG2b and IgG2c isotypes related to Th1 response (Figure 5d,e, *p* < 0.0007). Considering the values of the healthy rats plus two standard deviations as the negative cut-off, the proportion of asthmatic rats that developed anti-OVA antibodies was 100%, 100%, 100%, 89% and 78% for IgE, IgG1, IgG2a, IgG2b and IgG2c isotypes, respectively. Taking into account the serum dilution used in each assay, those antibodies belonging to IgG1 and IgG2a (Th2-associated) were more abundant than those belonging to the isotypes associated with a Th1 response (Figure 5a–c).

#### 2.2.4. IgE Antibodies and Leukotrienes in Bronchoalveolar Lavage Fluid

**Figure 6:**
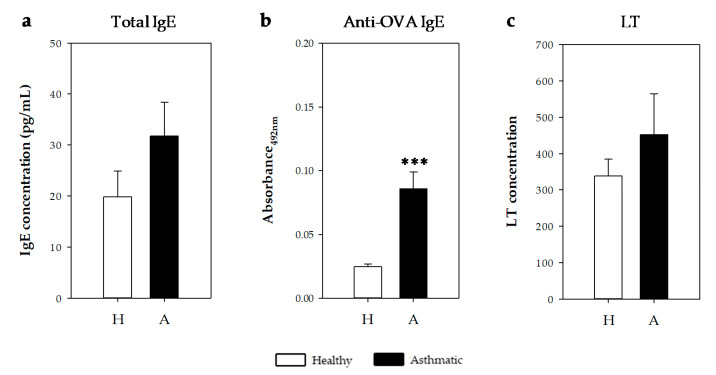
(**a**) Total and (**b**) anti-OVA IgE concentrations and (**c**) CysLT content in bronchoalveolar lavage fluid (BALF) samples obtained 24 h after the i.n. challenge in healthy (H, white bars) and asthmatic rats (A, black bars). Results are expressed as mean ± standard error (*N* = 9). Significant differences: *** *p* < 0.001 vs. healthy animals.

Anti-OVA IgE specific antibodies were also quantified in bronchoalveolar lavage fluid (BALF) samples obtained 24 h after the i.n. challenge (Figure 6). Asthmatic rats showed higher levels than healthy rats (Figure 6a, *p* < 0.0002). Considering the values of the healthy rats plus two standard deviations as the negative cut-off, 100% of asthmatic rats had anti-OVA IgE antibodies in BALF samples.

On the other hand, total IgE and cysteinyl leukotriene (CysLT) concentrations were quantified on BALF samples. IgE concentration tended to increase in the asthmatic animals (Figure 6b). Similarly, the CysLT concentration tended to increase in asthmatic rats’ BALF collected one day after the i.n. challenge (Figure 6c).

The correlation between BALF CysLT values and specific and total BALF IgE concentrations from the asthmatic animals was also studied. Anti-OVA IgE levels did not correlate with CysLT values. However, a significant positive correlation was found when considering the content of CysLT and total IgE in BALF samples (*r* = 0.833, *p* = 0.010).

#### 2.2.5. Cytokine and Leukocytes in Bronchoalveolar Lavage Fluid

**Figure 7:**
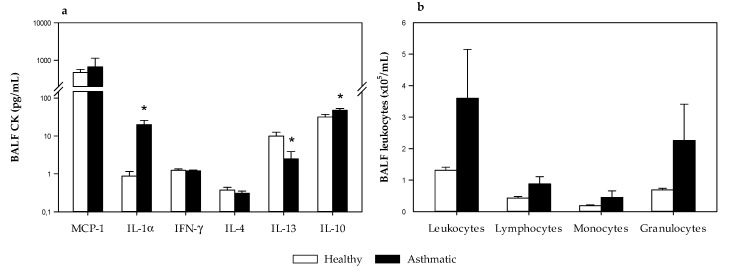
(**a**) Cytokine concentration and (**b**) leukocyte content in bronchoalveolar lavage fluid (BALF) samples obtained 24 h after the i.n. challenge in healthy rats (H, white bars) and asthmatic rats (A, black bars). Results are expressed as mean ± standard error (*N* = 9). Significant differences: * *p* < 0.05 vs. healthy animals.

The concentration of monocyte chemoattractant protein 1 (MCP-1), interleukin (IL) 1α, interferon (IFN) γ, IL-4, IL-13 and IL-10 were quantified in BALF samples obtained 24 h after the i.n. challenge (Figure 7a). MCP-1 and IL-1α are inflammatory cytokines, IFN-γ is associated with Th1 responses, and IL-4, IL-13 and IL-10 are associated with Th2 responses [23]. Although MCP-1 was the most abundant, IL-1α increased in the BALF of asthmatic animals (*p* < 0.05, Figure 7a). No changes were observed in IFN-γ and IL-4 concentrations. Nevertheless, BALF from asthmatic rats showed lower IL-13 levels and higher IL-10 concentrations than healthy rats (*p* < 0.05, Figure 7a).

On the other hand, leukocytes, lymphocytes, monocytes and granulocytes were also enumerated in BALF samples (Figure 7b). The number of leukocytes tended to increase in asthmatic rats without reaching statistical significance due to high variability among animals. Although the three types of cells tended to increase, granulocytes seemed to be mainly responsible for such increases.

## 3. Discussion

Studies in experimental models can contribute to understanding allergic asthma pathophysiology as well as to participating in the screening of new drugs and other therapeutic or preventive approaches. Different animal models of asthma have been available for many years. Specifically, rats have demonstrated similar features of airway allergy and allergic asthma to those exhibited by humans [7]. Here we introduce a rat model of allergic asthma that is easy to induce, in which anaphylactic shock can be objectively quantified by changes in motor activity and body temperature, among others.

In line with previous animal studies in asthma [18] and systemic and food allergy [17,19,24,25], we have used the Brown Norway rat strain because it is naturally atopic and, as in systemic and food allergy models, it is able to produce an IgE immune response to certain antigens. Moreover, female rats were chosen because at least in the case of Lewis rats, they show an exacerbated immune response [15].

In the preliminary design, it was demonstrated that Brown Norway rats need sensitization with *B. pertussis* toxin to produce a higher IgE response able to induce anaphylactic shock when an allergen is given by intranasal route, mimicking an asthmatic attack. Furthermore, this anaphylactic response was accompanied by changes in the nasal architecture the day after, as revealed by histological studies. In other studies in rat asthma models, authors applied *B. pertussis*, inactivated or not, together with intraperitoneal (i.p.) sensitization [26,27] or by an alternative route such as the foot pad [28]. Nevertheless, other studies did not apply an adjuvant [12,29,30]. However, in the present asthma model, sensitization with two i.p. administrations of OVA together with an i.n. challenge with a high dose of allergen was enough to produce anaphylactic shock. Therefore, it contrasts with other studies that required three or more i.p. administrations of OVA [29,31,32] and instillation of OVA nebulized by aerosol for several continuous or alternate days [27,33]. In addition, this first design evidenced the necessity of using approaches to objectively evaluate shock-associated symptoms.

The relationship between changes in rodent behavior and the immune response was well established years ago [34] and again recently [35]. Rodents with rhinitis show behavioral changes, such as anxiety-like behavior and reduced social interactions [36]. Moreover, anaphylaxis in mice induces scratching and rubbing around the nose and head, puffiness around the eyes and mouth, piloerection, increased respiratory rate, wheezing and decreased motor activity [37]. The evaluation of these symptoms requires the observation of each animal and this can be subject to personal interpretation. To quantify behavior changes, objective approaches should be used. In this sense, in previous studies focused on food allergy, the quantification of the decrease in motor activity after the induction of an oral challenge was objectively evaluated by activity meters [17,38]. The present study revealed that the i.n. challenge in sensitized rats could be evaluated by this approach, showing a decrease in the motor activity that remained at least until the day after the challenge.

Similarly, an anaphylactic response induced a decrease in body temperature, which must be a consequence of the systemic vasodilation produced by mast cell mediators [39]. Previous studies where animals were challenged orally, demonstrated a decrease of about 3 °C that remained for at least 2 h [17]. Herein, the body temperature achieved its minimum value 3 h after the challenge and, interestingly, it remained low even 20 h after shock induction. It is worth highlighting the use of a sensitive method to measure the temperature, such as the loggers placed in the peritoneal region of each animal one week before the challenge. Such an approach has been previously used to study the effect of the circadian rhythms in rats [40]. Although there are many studies in which rat asthma models are applied for the screening of drugs or other therapeutic approaches, anaphylactic shock has not been quantified in vivo [33,41].

Major variables quantified in animal models include IgE, cytokines, histopathological features and some functional tests [11,26,27,29,30,31]. In this sense, the i.p. sensitization by OVA and one booster 7 days later induced the synthesis of specific IgE and IgG antibodies in sera. Previous studies quantified specific IgE in asthmatic rats [27], however specific IgG is often not included. Our results show that asthmatic rats produced higher amounts of anti-OVA antibodies belonging to IgG1 and IgG2a classes than those belonging to IgG2b and IgG2c isotypes. These results meet the criteria established by Knippels et al. which stated that a good animal model should produce a significant amount of IgE- and/or other Th2-related antibodies [25]. It has been demonstrated that IgG1 and IgG2a are isotypes related to Th2 response in rats [17,21,22]. Therefore, this model will be closer to the type 2 asthma endotype, including the most common phenotypes, which involves Th2 cells, IgE-producing B cells, group 2 innate lymphoid cells, IL-4-producing natural killer (NK) cells, NK-T cells, basophils, eosinophils, mast cells and their major cytokines [42]. In fact, when analyzing the leukocytes present in the BALF, a tendency to increase the content of granulocytes that would include eosinophils was observed. Further experiments focused on this particular leukocyte should be carried out. On the other hand, the BALF cytokine profile revealed that the inflammatory cytokines such as IL-1α increased, whereas typical Th2-related cytokines such as IL-4 and IL-13 did not increase. These results need to be confirmed and completed by determinations not just 24 h after the shock and, for example, with cytokine gene expression in the lung.

In addition, total and specific IgE concentrations were measured in BALF. Although anti-OVA IgE antibodies were found in all OVA-sensitized rats, the quantification of total IgE did not reveal a significant increase. It could be due, at least in part, to the high variability between rats in the same group. Similarly, when quantifying the CysLT concentration in BALF, values did not achieve statistical significance. CysLT are early mediators of asthma in rats [7] that can be secreted by eosinophils and CysLT may be involved in the accumulation of eosinophils in the airways of asthmatics [42]. We found a positive correlation between CysLT and total IgE in the BALF, meaning that the higher the LT concentration in rats, the higher the total BALF IgE content. Such mediators are important in the pathophysiology of asthma [42]. Further studies should be carried out to better characterize the cells, i.e., eosinophils and mast cells, and the mechanisms involved in our asthma model.

## 4. Materials and Methods

### 4.1. Animals and Ethical Issues

Four-week-old female Brown Norway rats were obtained from Envigo (Huntingdon, UK) and housed in polycarbonate cages containing bedding of large fibrous particles (Souralit 1035, Bobadeb S.L., Santo Domingo de la Calzada, Spain) with two or three rats per cage under controlled conditions of temperature and humidity in a 12:12 h light/dark cycle. The animals had unrestricted access to OVA-free food (American Institute of Nutrition 93M formulation, AIN-93M, Envigo Teklad Diets, Madison, WI, USA) and water and remained in one week of quarantine before experiments began.

With regard to sample size estimation, the Appraising Project Office’s Program from the Universidad Miguel Hernández de Elche (Alicante, Spain) was used to calculate the minimum number of animals providing statistically significant differences among groups, assuming no dropout rate and type I error of 0.05 (two-sided).

The animal procedures were in accordance with the institutional guidelines for the care and use of laboratory animals and approved by the Ethical Committee for Animal Experimentation of the University of Barcelona and the Catalonia Government (CEEA/UB ref. 414/16 and DAAM 9351, respectively), in full compliance with national legislation following the EU-Directive 2010/63/EU for the protection of animals used for scientific purposes.

### 4.2. Experimental Designs

A preliminary experimental design was carried out in order to study whether *B. pertussis* toxin (Bpt) was required for allergy induction and if the conditions tested were sufficient to induce an anaphylactic response by intranasal (i.n.) route (Figure 1a). For this, on day 0, rats were sensitized by an i.p. injection of a suspension (500 µL) containing 50 µg ovalbumin (OVA, grade V, Sigma-Aldrich, Madrid, Spain), 20 mg alum (Imject®, Pierce, IL, USA) with or without 50 ng Bpt (Sigma-Aldrich). At day 7, a booster was given to rats by i.p. injection of 500 µL of a suspension containing 50 µg OVA in 20 mg alum. At day 27, blood was collected from the saphenous vein to test the specific IgE production. As all rats synthesized anti-OVA IgE, an i.n. challenge with 300 µL (drop by drop in two charges of an automatic pipette of 200 µL in the immobilized animal) of either 5 or 50 mg/mL OVA was carried out at day 29. Anaphylactic shock was then assessed by changes in rat behavior, motor activity and body temperature. One day later, animals were euthanized and nose tissue was collected.

After considering the results of the preliminary study, a definitive design was carried out and the anaphylactic shock was quantified by unbiased measurements and for a longer period (Figure 1b). In this second round of experiments, rats were sensitized with 50 µg OVA, 20 mg alum and 50 ng Bpt (i.p.) and boosted a week later with 50 µg OVA in 20 mg alum (i.p.). A parallel group of non-sensitized rats (age and sex matched) was included. At day 28, all rats received an i.n challenge with 300 µL of 50 mg/mL OVA. Anaphylactic response was accurately assessed by changes in motor activity and body temperature. Blood and BALF samples were obtained 24 h later.

### 4.3. Motor Activity Assessment

In the preliminary study, changes in motor activity were assessed by observation in a blinded manner of a video recorded from each animal (isolated in a cage) before and immediately after the i.n. challenge for 10 minutes.

In the definitive study, the movements of each animal were quantified by an activity meter as previously performed [38]. Briefly, the animals were placed in an isolated room and housed in small cages individually. Motor activity was recorded using activity-meters consisting of two perpendicular infrared beams. Every time the animal crossed one beam a count was detected. Number of movements was recorded every hour from 4 h before the i.n. challenge (basal) until 20 h after shock induction.

### 4.4. Temperature Measurement

In the first design, body temperature was measured prior to the i.n. challenge, every 5 min for 15–30 min after the challenge by a rectal probe (Oakton Acorn Temp J-K-T Thermocouple Thermometer WD-35627-00, Physitemp Instruments, Oakton, Vernon Hills, IL, USA). As the results obtained with this thermometer did not have enough sensitivity to quantify the decrease in body temperature induced by the shock, in the second round of experiments body temperature was recorded by data loggers (Thermochron^®^, iButton type DS1921H-FS with a resolution of 0.125 °C). For this, one week before the i.n. challenge, a logger was intraperitoneally implanted into each rat under isoflurane (Isoflo®) anesthesia (4%–5% in the induction and 1%–2% in the maintenance, with an oxygen flow of 0.5–1.0 L/min). Animals were then isolated in individual cages. Meloxicam (1 mg/kg BW, subcutaneous route) was administered immediately after the intervention and 24 h later. Body temperature was recorded every 10 minutes from 4 h before the challenge until 20 h later. Body temperature in the period after challenge was summarized as the AUC considering changes above 34 °C.

### 4.5. Blood Collection

In the first experiments, blood samples were collected from the saphenous vein before immunization to be used as non-sensitized reference samples. Blood collection was repeated 4 weeks later before the i.n. challenge.

In the definitive design, one day after the i.n. challenge rats were anaesthetized with ketamine/xylazine (90 and 10 mg/kg, respectively; Merial laboratories S.A., Barcelona, Spain; Bayer A.G., Leverkusen, Germany). Blood was collected by heart puncture and serum was kept at −20°C until anti-OVA antibody quantification.

### 4.6. BALF Collection

After exsanguination, the trachea was exposed by surgically opening the neck region with minimum incisions possible. Five mL of chilled phosphate buffered saline (PBS) was instilled into the lung using a 10 mL syringe fitter with a 16G catheter. The PBS was allowed to stay in the lungs for 30 s, while the thoracic area was gently massaged and BALF was retrieved and instilled again with the help of a syringe. This was repeated three times. The procedure was done again to a final volume of 10 mL PBS. The BALF was centrifuged at 538× *g* at 4 °C and the supernatant was collected and stored either at −80 °C or −20 °C for further analysis.

### 4.7. IgE and Anti-OVA Antibodies Quantification

IgE concentration in BALF samples was quantified by a sandwich enzyme-linked immunosorbent assay (ELISA) using anti-rat IgE antibody as the capture reagent and biotin-conjugated anti-rat IgE antibody as the detection reagent [17,24]. Undiluted BALF samples were added, while IgE standard was added in a concentration range of 0.15–20 ng/mL.

Specific IgE antibody isotype in serum and BALF samples were quantified using an antibody-capture ELISA as previously performed [17,24]. Results are shown as absorbance units obtained from all samples analyzed in the same ELISA plate. Serum samples were diluted 1/10, whereas BALF samples were processed undiluted.

The serum concentration of anti-OVA total immunoglobulin concentrations as well as IgG1, IgG2a, IgG2b and IgG2c isotypes were assessed by ELISA as previously described [17,24]. Results are shown as absorbance units obtained from all samples analyzed in the same ELISA plate. For serum anti-OVA IgG1 and IgG2a quantification, samples were diluted at 1/400,000 and for serum IgG2b and IgGc, samples were diluted 1/1,600 and 1/400, respectively.

### 4.8. Histological Study

Noses were excised and set overnight in 4% buffered formaldehyde at room temperature. Then, fixed nose tissues were dehydrated, embedded in paraffin and cut into 6 µm-thick sections using a microtome (RM2135, Leica, Wetzlar, Germany). Subsequently, the sections were stained with hematoxylin-eosin and mounted on glass slides. The observation of the nasal architecture was performed using a bright-field microscope (Olympus BX41, Olympus Corporation, Shinjuku, Tokyo, Japan) and an Olympus XC50 camera (Olympus, Tokyo, Japan) at 100×.

### 4.9. Quantification of Cysteinyl Leukotriene (CysLT)

The concentration of CysLT in BALF was quantified using a cysteinyl leukotriene ELISA kit (Enzo Life Sciences Inc., New York, USA) in accordance with the manufacturer’s instructions. Extraction of LTs was first carried out by sample acidification (with 2 M HCl) to pH 3.5 and concentration through a C18 reverse-phase column following the manufacturer’s instructions.

### 4.10. Quantification of Cytokines

The concentration of MCP-1, IL-1α, IFN-γ, IL-4, IL-13 and IL-10 was quantified in BALF samples using a ProcartaPlex^®^ multiplex immunoassay (eBioscience). The concentration of each cytokine was obtained by MAGPIX^®^ analyzer (Luminex Corporation, Austin, TX, USA) at the Cytometry Service of the Scientific and Technological Centers of the University of Barcelona (CCiT-UB). Assay sensitivity was as follows: 15 pg/mL for MCP-1, 10 pg/mL for IL-1α, 0.62 pg/mL for IL-4, 3 pg/mL for IL-13 and 6.01 pg/mL for IL-10.

### 4.11. Leukocytes in BALF

An aliquot of BALF samples was concentrated by centrifugation and was then analyzed with an automated hematology analyzer (Spincell, MonLab Laboratories, Barcelona, Spain) calibrated for rat leukocytes. Total leukocytes, lymphocytes, monocytes and granulocytes were quantified and expressed as cells/mL BALF after considering the conditions of sample concentration.

### 4.12. Statistical Analysis

The Statistical Package for the Social Sciences (SPSS v22.0, IBM, Chicago, IL, USA) was used for statistical analysis. Data were tested for homogeneity of variance and normality distribution by the Levene’s and Shapiro–Wilk tests, respectively. Non-parametric Mann–Whitney U and Friedman tests were used in order to assess significance for independent and related samples, respectively.

To explore the functional correlation between the antibody and CysLT production, body temperature and motor activity changes, Spearman´s correlation analyses were performed considering values from the asthmatic group.

## 5. Conclusions

In conclusion, by means of a combination of two i.p. immunizations (the first one with *B. pertussis* toxin), we have established an easy and effective rat model of allergic asthma in female Brown Norway rats that induces the synthesis of specific Th2-related antibodies, especially IgE, and therefore an anaphylactic response after i.n. challenge. Moreover, the anaphylactic shock can be unbiasedly quantified by changes in motor activity and body temperature, which remained for at least 20 h after the i.n. challenge. Apart from the anaphylactic response, further experiments should be carried out to establish other functional consequences such as vascular and lung responses, as well as the mechanisms involved in such models. Further experiments should also assess cells and mediators involved, such as eosinophils and mast cells and their products. This model, which could potentially be widely applied to other aeroallergens and always administered with alum and *B. pertussis* toxin, could be useful for studying both therapeutic and dietetic interventions in order to prevent or treat this prevalent disease.

## Abbreviations

AUC	Area under the curve
BALF	Bronchoalveolar lavage fluid
Bpt	*Bordetella pertussis* toxin
CysLT	Cysteinyl leukotriene
ELISA	Enzyme linked immunosorbent assay
IL	Interleukin
i.n.	Intranasal
IFN	Interferon
i.p.	Intraperitoneal
MCP-1	Monocyte chemoattractant protein 1
OVA	Ovalbumin
Th	T helper
